# Polyhydroxyalkanoate (PHA) Enriched with Diatomaceous Earth as a Sustainable Ecological Composite Material with the Possibility of Machining

**DOI:** 10.3390/ma18245571

**Published:** 2025-12-11

**Authors:** Anna Gaweł, Andrzej Matras

**Affiliations:** Department of Production Engineering, Faculty of Mechanical Engineering, Cracow University of Technology, 31-864 Kraków, Poland; anna.gawel@pk.edu.pl

**Keywords:** polyhydroxyalkanoates, organic fillers, diatomite, diatomaceous earth, PHA, ecological composites, cutting, milling, surface roughness, cutting forces, chip temperature

## Abstract

**Highlights:**

**What are the main findings?**

- Use of organic materials in the production process of polymer composites.
- Enabling the characterization of milling parameters of modern, ecological materials enriched with organic filler.
- Reducing the amount of environmental waste.

**What is the implication of the main finding?**

- No effect of diatomaceous earth content on the machinability of the analyzed composites was observed.
- Excessive temperature in the cutting zone may cause superficial melting, which deteriorates surface quality.
- During machining, the values of the feed component of the cutting force were below 11N.

**Abstract:**

In this study, the milling process of eco-friendly polymer composites enriched with an organic filler was analyzed. Polyhydroxyalkanoate was filled with 0.5%, 1%, and 2% of diatomite and produced via injection molding. Then, the milling process was performed on the obtained samples to determine the effect of diatomite content on the machinability of the produced composites. The results showed that the analyzed diatomite content in the machined samples had no significant influence on the cutting process. If the cutting parameters are not properly selected, excessive heat generated during machining can lead to a heterogeneous geometric surface microstructure. The milling process resulted in a series of high-quality surfaces (Ra < 2 μm), chip temperatures below 90 °C, and a feed component of the total cutting force below 11 N.

## 1. Introduction

Modern biobased polymers are produced from various sources [1]. The first group consists of materials produced from animal sources such as polybutylene succinate (PBS) [2], polylactic acid (PLA) [3], and polyhydroxyalcanoate (PHA) [4]. The second group consists of plant-derived materials such as starch, cellulose-based polymers, and alginate [5,6]. It is also possible to obtain biobased materials derived from bacterial fermentation products, which include collagen, chitin, and chitosan [7,8,9,10]. Another is a group of polymers produced in a specific way, including sericin, which is a by-product of the silk processing process [11]. Polymeric materials are increasingly used due to their price, weight, and strength properties [12]. The desire to minimize prices and reduce carbon dioxide emissions is causing a growing trend to produce ecological composite materials enriched with natural fillers [1,13].

Two subgroups of materials have been distinguished for polyhydroxyalkanoates. The first group includes Short-Chain Length polyesters (PHA SCL), which have from 3 to 5 carbon atoms in the monomers. The second group, called medium-chain monomers (PHA MCL), contain from 6 to 16 carbon atoms [14]. One of the most well-known polyhydroxyalkanoates is polyhydroxybutyrate. Despite its thermoplasticity, it is also a brittle and stiff polymeric material [15]. The properties of this polymer are due to its high crystallinity [16]. Typically, attempts are made to change its mechanical properties by creating copolymers of hydroxyvalerate with hydroxybutyrate. Adding 30% 3HV to PHB reduces the Young's modulus and glass transition temperature. This results in thermoplastic properties of the polymer, which facilitates its processing [17].

Biogenic debris accumulates at the bottom of lakes and other aquatic ecosystems in the form of siliceous algae to carbonate precipitates [18]. Diatoms are called unicellular algae, forming a shell or frustule. They accumulate in large quantities in sediments, where they fossilize to form diatomite [19]. This is a friable sedimentary rock with a SiO_2_ diatom content of at least 50%. It was assumed that a sediment in which the porosity is over 70% and the diatom content is at least 50% is a diatomaceous mud if it is not consolidated, and it is diatomite if it is consolidated. As temperature and burial depth increases, porosity is lost and may result in recrystallization into porcelanite, flint, and pure quartz [18]. Diatomite is a natural porous biomass material that is characterized by porosity, low bulk density, stable chemical properties, and large surface area [20].

Mei et al. prepared composite materials based on polylactic acid (PLA) and poly(butylene adipate-co-terephthalate) (PBAT) in order to increase the antimicrobial resistance of ZnO-based materials by enriching them with diatomite. They showed that diatomaceous earth has a higher antibacterial activity than zinc oxide [21]. Zhu et al. produced a composite consisting of hybrid sisal fibers (HSF), polylactide, and diatomite. The addition of diatomaceous earth improved the composites’ mechanical and thermal properties [22]. Dobrosielska et al. demonstrated that the addition of synthetic wax to polylactide composites modified with diatomaceous earth, three types of silanes, and natural and synthetic waxes increased the tendency of the diatomaceous earth to agglomerate. The natural wax influenced the proper dispersion of the filler in PLA matrix [23]. Materials based on polylactic acid (PLA) and polybutylene succinate (MAPBS) grafted with maleic anhydride have also been developed. Diatomite, which absorbs polyethylene glycol (PEG), has also been added to create a thermostatic and biodegradable composite [24].

Many researchers also analyze the cutting of plastics and their composites. Ciecieląg [25] milled thin-walled polymer composites with glass fiber and carbon fiber saturated with epoxy resin. They performed machining to ensure dimensional accuracy and adequate surface quality, observing adverse effects of increasing the feed rate. The authors of [26] also milled thin-walled components made of polymer composites. They noted that when machining Carbon Fiber Reinforced Polymer (CFRP) composites, the effect of feed rate on surface roughness is less pronounced than, for example, when machining aluminum alloys. Doluk et al. [27] used a diamond-coated tool to machine a two-layer composite of 2024 aluminum and CFRP. They noted differences in the roughness parameters and surface topography of individual composite layers. Bílek et al. [28] investigated the effectiveness of adaptive trochoidal milling in the machining of glass fiber-reinforced polymers. Using a four-flute cutter with a helix angle of 10° resulted in the best performance, minimal tool wear, and high surface finish (Ra = 1.36 μm). Xu and Wang [29] milled CFRP composites with Polycrystalline Diamond (PCD) tools. They investigated the effect of cutting parameters and tool rake angle on cutting force and surface roughness. The results showed that a PCD tool with a 4° rake angle had lower cutting forces than a PCD tool with a 0° rake angle, and the effect of rake angle on surface roughness is negligible. Yan et al. [30] machined three thermoplastic polymers: polymethyl methacrylate, polyetheretherketone, and polyimide. They indicated that thermoplastic polymers are considered among the most promising materials for the future. Therefore, further research into their mechanical properties and machinability is necessary, especially in ultra-precision machining. When machining polyamide at high cutting speeds and high feed rates, lower temperatures are observed in the cutting zone.

Cutting biodegradable materials is a modern field of mechanical engineering. Lack of data on the effect of diatomite on machining parameters means this remains an unexplored topic requiring further research. Diatomite and polyhydroxybutyrate (PHA) are biocompatible materials, making them widely used in tissue engineering and implants. The specific shapes of such components make milling the material more cost-effective than designing a single mold. The main difference is that previous research has been based on already-known polymeric materials or composites. The goal of our work was to create an eco-friendly material, previously unknown, whose matrix and filler are biocompatible. Both diatomite and polyhydroxyalkanoates are biocompatible and biodegradable, making them perfectly suited for this application. Due to their specific shapes, tissue engineering and implants require adapting shapes and geometries to specific requirements. This means that additional machining will likely be required. It was planned to use the machining method, which will be suitable for this purpose because the addition of diatomite has no effect on the composite's machinability.

This study analyzed the machinability of composites consisting of a PHA matrix and various percentages of diatomite. The milling process of a groove with a width equal to the full diameter of the tool was analyzed. The effect of feed rate was tested. Section 2 discusses the method of composite production, research, and equipment technique. Section 3 discusses the effects of the tested parameters on the obtained chip temperature values, the value of the feed component of the cutting force, and the surface roughness. A statistical analysis of the results and a discussion are presented. Section 4 summarizes the obtained results.

## 2. Materials and Methods

### 2.1. Materials and Composite Preparation

The matrix for produced composites was polyhydroxyalkanoates (PHA) from Natureplast (Mondeville, France) under the trade name PHI 002 for injection molding. Two groups of composites were produced: pure polyhydroxyalkanoates and polyhydroxyalkanoates as a matrix with appropriate weight percentage. Samples with top surface dimensions of 20 mm × 25 mm were produced using a KraussMaffei 30-125 C injection molder (KraussMaffei Group GmbH, Parsdorf, Germany). Before processing, PHA pellets were dried in DRYMAX primus E30-70-M molecular dryer (Wittmann Battenfeld GmbH, Kottingbrunn, Austria) at 40 °C for 4 h. This step was implemented to removed water. A milling process was also carried out on samples after the injection molding process to determine the optimal properties. The basic parameters used during sample production are presented in Table 1.

Diatomaceous earth, used as a biodegradable waste material, has beneficial effects on lowering carbon dioxide emissions, reducing production costs, and accelerating environmental degradation processes. The resulting composites form a broad category of High Green Polymeric Composites (HGPC) materials. Consequently, the visual properties of the produced materials also improved, expanding their potential applications. The chemical compositions are shown in Table 2.

### 2.2. Milling of Ecological Composites

A numerically controlled MiniMIll2 (Haas, Oxnard, CA, USA) milling machine was used to process prepared composites samples. Machining was carried out using a four-flute end mill (Seco Tools AB, Fagersta, Sweden) with a diameter of 12 mm and catalogue designation 880120R020Z4.0. The cutting tool was made of tungsten carbide and coated with a polycrystalline diamond layer under the trade name DURA. The cutter is shown in Table 3. It is designed for machining non-metallic materials, ensuring maximum productivity. The parameters of used cutting tool are summarized in Table 3.

During the tests, a 12 mm groove was made along the entire length of each prepared sample. Before testing, the top surface of the sample was pre-machined, and the mounting was not changed afterwards. Constant machining parameters were used: single machining pass, a cutting depth of 0.5 mm, a cutting width of 12 mm, a cutting speed of 150 m/min, a spindle speed of 3980 rev/min, and a variable feed rate in the range of 0.05 mm/t to 0.13 mm/t. The cutting parameters were selected based on the manufacturer's recommendations and previously performed preliminary experimental tests. A spindle warm-up procedure was performed before testing. This allowed for a stable tool temperature during tests. Between each test, the tool temperature was allowed to stabilize, and the tool and the workpiece was inspected. The tests were performed at a stable ambient temperature of 22 °C. No tool wear or adhesion of the workpiece material to the tool surface was observed during the tests.

The effects of two variables were tested. The experimental studies were conducted based on a complete test plan in round robin combinations, resulting in 12 cases included in the research plan. Each test was repeated four times, which yielded a total of 48 experimental datasets in this study. Samples were tested in random order. The analyses were conducted based on a significance level of α = 0.5. Linear effects were analyzed. Table 4 summarizes the tested feed rates and diatomite content in the samples.

In Table 5, the basic properties of the polyhydroxyalkanoate produced via injection molding are presented.

### 2.3. Characterization of Measuring Equipment

A thermal imaging camera was used to record and analyze the temperature field distribution in the cutting zone, FLIR SC 620 (Teledyne FLIR, Wilsonville, OR, USA), with an integrated fixed focal length lens equal to f = 38 mm. Thermogram analysis was performed using dedicated software, FLIR Thermal Studio 2.0.58.0. Thermograms were recorded at a resolution of 640 × 480 px with a frequency 30 Hz. During tests, the experimentally determined emissivity was set to 0.90. The camera was placed at a distance of 0.8 m from the observed sample, and the measurement range was from 0 to 120 °C.

A piezoelectric dynamometer was used to measure the feed component of total cutting force, Kistler 9257B (Kistler, Winterthur, Switzerland). Samples were attached using a clamping arm. The dynamometer was connected to a charge amplifier, Kistler 5070B. The obtained force values were analyzed using the software Kistler DynoWare, version 2825A.

The surface roughness was measured using a profilometer Talysurf Intra 50 (Taylor Hobson, Leicester, Great Britain). Measurements were taken in the feed direction. The measuring tip with a 2 μm radius was used to measure the surface roughness. Surface roughness parameters were calculated based on the standard ISO 4287 [31]. The cut-off wavelengths were set to λc = 0.8 mm and λs =2.5 μm. Measurements were performed on a length of 5 mm.

Microscopic observations of surfaces were performed using an optical microscope, VHX-600 (Keyence, Osaka, Japan) with magnification 200×.

## 3. Results and Discussion

### 3.1. Temperature Analysis in Cutting Zone

**Figure 1:**
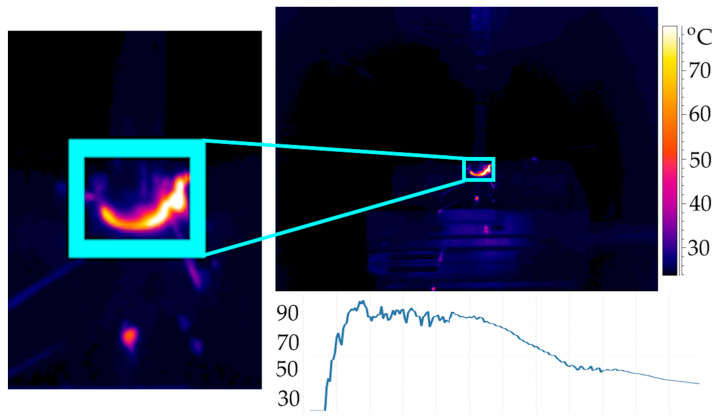
Thermogram recorded during machining simultaneously with an example of the maximum chip temperature waveform for sample with 1% of diatomite and f_z_ = 0.05 mm/t.

During machining, maximum chip temperature measurements were taken. The average value was determined from the maximum temperature values read within the frame shown in Figure 1. The values recorded during stable processing were analyzed. One measurement was performed for each sample, yielding 48 data points.

**Figure 2:**
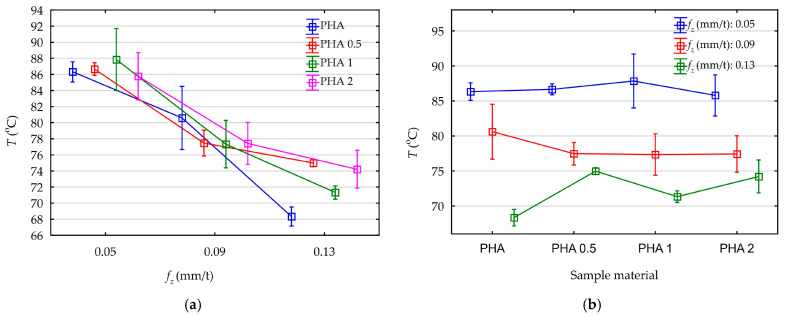
The influence of feed rate and diatomite content on the average values of chip temperatures: (**a**) feed rate influence; (**b**) the influence of diatomite content.

In Figure 2a,b, the observed influence of feed and diatomite content in the samples based on average values of chip temperature along with their standard deviations are presented.

Analyzing Figure 2a,b, the effect of feed on chip temperature values is clearly visible. The effect of diatomite content in the samples is within the standard deviations. This makes it insignificant from the point of view of machining technology. Without taking into account the diatomite content in the samples, the corresponding temperatures observed were amounted appropriately:

- f_z_ = 0.05 mm/t, T_mean_ = 86.65 °C,
- f_z_ = 0.09 mm/t, T_mean_ = 78.20 °C,
- f_z_= 0.13 mm/t, T_mean_ = 72.21 °C.

**Figure 3:**
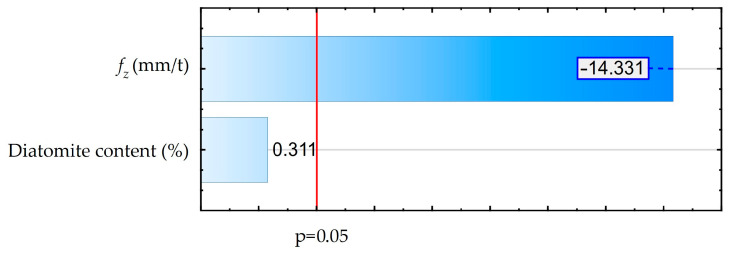
Pareto diagram for the influence of feed rate and diatomite content on chip temperature.

The values indicate an unusual heat balance for the cutting process of the analyzed materials. Increasing the volume of the cutting layer does not lead to a dominant effect of higher heat release. Probably, due to its low mechanical strength, the amount of heat released as a result of material deformation in the cutting zone is not dominant. However, this assumption is based only on analysis of temperature trends, and full proof requires analysis of the energy balance. Similar trends have also been observed by other researchers, but in no case is an energy balance analysis performed [30,32,33,34]. In order to determine the statistical effect of feed and diatomite content on chip temperature values for the assumed confidence level of 95% (*p* = 0.05), a Pareto diagram was made, as shown in Figure 3. An ANOVA was also performed. These results are shown in Appendix A (Table A1), and both analyses are consistent.

In Figure 3, the red vertical line denotes the significance threshold corresponding to the chosen confidence level. Parameter values below this line are considered statistically insignificant. The labels on the bars indicate the numerical values of the standardized effects. The performed analysis showed a significant effect of feed. Simultaneously, the effect of diatomite content is statistically insignificant.

### 3.2. The Feed Component of the Total Cutting Force

**Figure 4:**
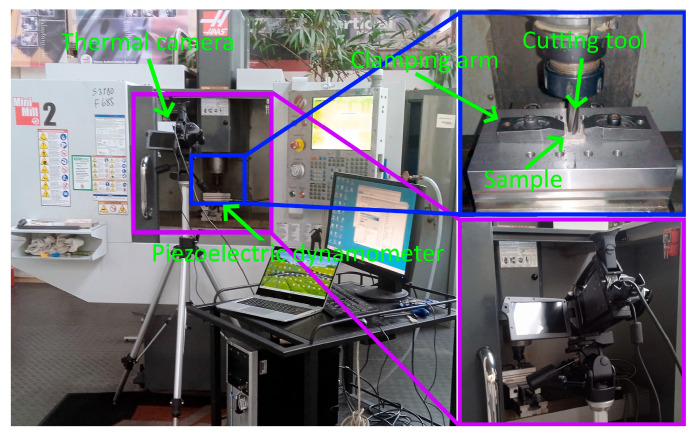
The view of the assembled test stand and the method of mounting the sample on the dynamometer.

During the milling of prepared samples, the values were measured simultaneously with the chip temperature measurements of feed component F_f_ of total cutting force. One measurement was performed for each of the samples. The values of the feed component of total cutting force for each sample were established by determining the average value of the data recorded during the measurement and following a stable course of the analyzed force. Therefore, 48 data points were obtained, 4 for each point of the research plan. The view of the assembled test stand and the method of mounting the sample on the dynamometer is shown in Figure 4.

**Figure 5:**
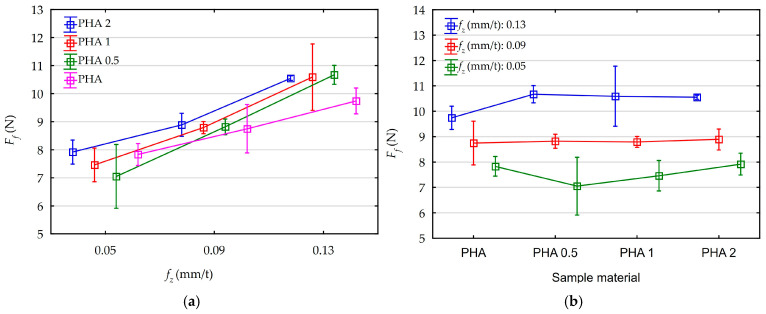
Feed rate and diatomite content influences on the recorded average values of feed component and total cutting force: (**a**) feed rate influences, (**b**) diatomite content influences.

Figure 5 shows the influence of feed rate and diatomite content on the average values of feed component of total cutting force along with their standard deviations.

Analyzing the above graphs, the influence of feed on the values of the feed component of the total cutting force is clearly visible. Moreover, all observed average values of the analyzed cutting force are in a range below 11 N. Without taking into account the diatomite content in the samples, the following force values were obtained for the individual parameters:

- f_z_ = 0.05 mm/t, F_f_ = 7.57 N,
- f_z_ = 0.09 mm/t, F_f_ = 8.81 N,
- f_z_ = 0.13 mm/t, F_f_ = 10.39 N.

Taking into account the diatomite content in the samples (regardless of the feed rate), the average values are as follows:

- PHA, F_f_ = 8.78 N,
- PHA 0.5, F_f_ = 8.85 N,
- PHA 1, F_f_ = 8.95 N,
- PHA 2, F_f_ = 9.12 N.

**Figure 6:**
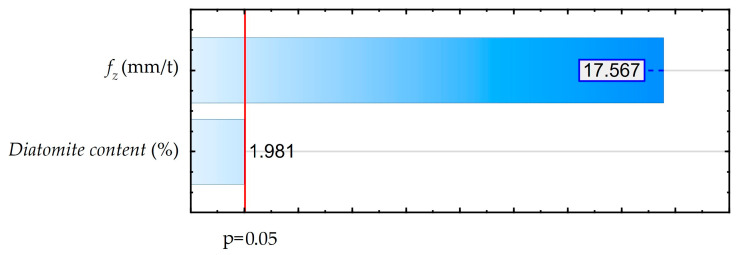
Pareto diagram for the influence of feed rate and diatomite content on the feed component of the total cutting force.

As shown in Figure 6, a Pareto diagram was made to determine the statistical effect of feed and diatomite content on the component of total cutting force for assumed confidence level of 95% (p = 0.05). An ANOVA was also performed. These results are shown in Appendix A (Table A2). The results of both analyses are consistent.

The analysis performed showed a significant effect of feed, while the effect of diatomite content is statistically insignificant. The diatomite content in the samples and the changes in the feed rate are irrelevant for the machining process. The recorded values of feed components of total cutting force are small. Machining of the tested materials involves low cutting resistance and low energy requirements.

### 3.3. Surface Roughness Analysis

**Figure 7:**
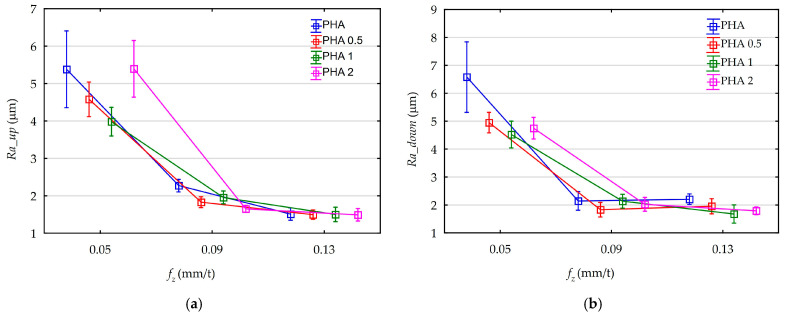
The influence of feed rate on the Ra parameter value of surface roughness: (**a**) up-milling area; (**b**) down-milling area.

As a result of measurements, the Ra surface roughness parameter was analyzed. Measurements were taken at the bottom of the groove. While milling grooves with the full tool diameter, two areas can be distinguished. These are delimited by the axis of symmetry of the machined surface. The first is located below the axis, in the area where down milling occurs. The second occurs above the axis and was characterized by up-milling kinematics. These two cutting kinematics have a significant influence on the surface roughness; therefore, separate measurements and analyses were also performed for both areas. Measurements of the Ra surface roughness parameter on each of four samples made for one point of the test plans were repeated four times: two measurements for down-milling area and two for the up-milling area. Therefore, 192 data points were analyzed to determine the effect of analyzed feed rates and diatomite content on the surface roughness Ra parameter. The influence of feed on the mean values of the surface roughness parameter Ra, along with their standard deviations obtained from the measurements, are shown in Figure 7a,b.

**Figure 8:**
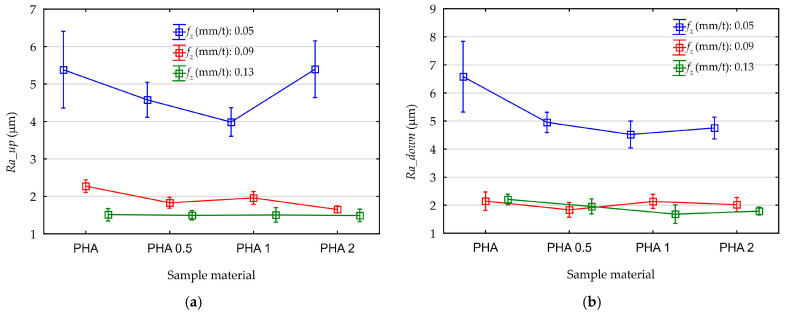
The influence of diatomite content on the Ra parameter value of surface roughness: (**a**) up-milling area; (**b**) down-milling area.

The effects of diatomite content on the mean values of the surface roughness parameter Ra along with their standard deviations are shown in Figure 8a,b.

Analyzing the above graphs, a dominant effect of feed on surface roughness is noticeable. The influence of process kinematics for both down-cut and up-cut milling on surface roughness is also observable. Characteristic areas of up-cut milling, regardless of the diatomite content in the sample, are characterized by higher surface roughness. The effect of feed on roughness compared to down-milling areas is as follows:

- f_z_ = 0.05 mm/t, Ra increase 15%,
- f_z_ = 0.09 mm/t, Ra increase 5%,
- f_z_ = 0.13 mm/t, Ra increase 25%.

As the feed rate increases, surface roughness decreases. This is particularly noticeable when increasing the feed rate from 0.05 mm/t to 0.09 mm/t. In this case, a 145% decrease is noticeable. Changing the feed rate from 0.09 mm/t to 0.13 mm/t causes a further 15% reduction in the average Ra parameter.

Analysis of effect of diatomite content in samples on surface roughness does not lead to such clear conclusions as in the case of feed and cutting kinematics. Analyzing the average values of the Ra parameter for all samples without categorization due to the feed, the following average results were obtained:

- PHA, Ra = 3.35 μm,
- PHA 0.5, Ra = 2.77 μm,
- PHA 1, Ra = 2.63 μm,
- PHA 2, Ra = 2.85 μm.

Without taking into account the high Ra values obtained for samples machined at a feed rate of 0.05 mm/t in the calculations, the average values are as follows:

- PHA, Ra = 2.03 μm,
- PHA 0.5, Ra = 1.77 μm,
- PHA 1, Ra = 1.81 μm,
- PHA 2, Ra = 1.73 μm.

**Figure 9:**
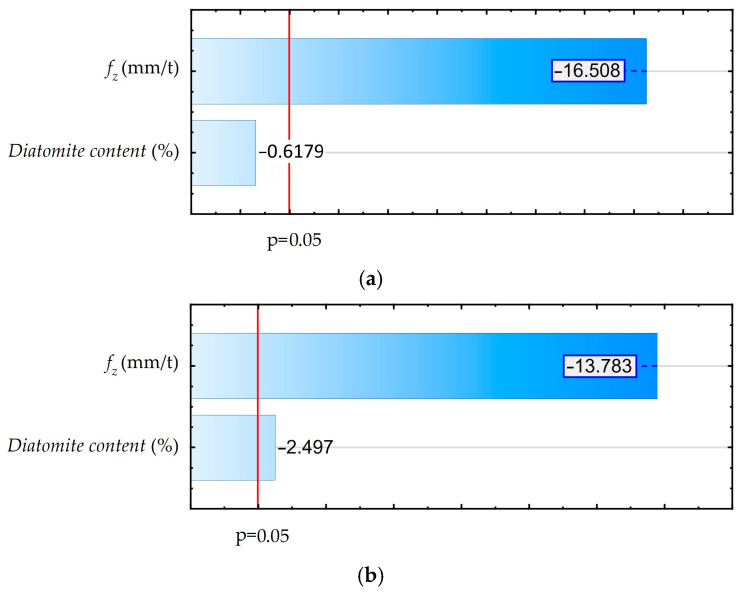
Pareto diagrams: (**a**) up-milling area; (**b**) down-milling area.

In order to determine the statistical effect of diatomite addition and feed rate changes for a confidence level of 95% (*p* = 0.05), Pareto diagrams were created, as shown in Figure 9. An ANOVA was also performed. Its results are shown in Appendix A (Table A3 and Table A4). The results of both analyses are consistent.

Analyzing the presented Pareto diagram, in the area of up-milling, a statistically significant effect of feed and an insignificant effect of diatomite addition on the values of the Ra parameter were obtained. In the area of down-milling, both the influence of feed and the addition of diatomite are significant. Pareto diagrams performed for the results without division into the down-cut and up-cut milling areas also indicate a statistically significant effect of feed and diatomite addition. Diatomite content is negligible on the surface roughness of the obtained samples. The average Ra values calculated for all analyzed feed rates were close to 0.72 µm. For feeds of 0.09 mm/t and 0.13 mm/t, the scatter data results were close to 0.2 µm.

**Figure 10:**
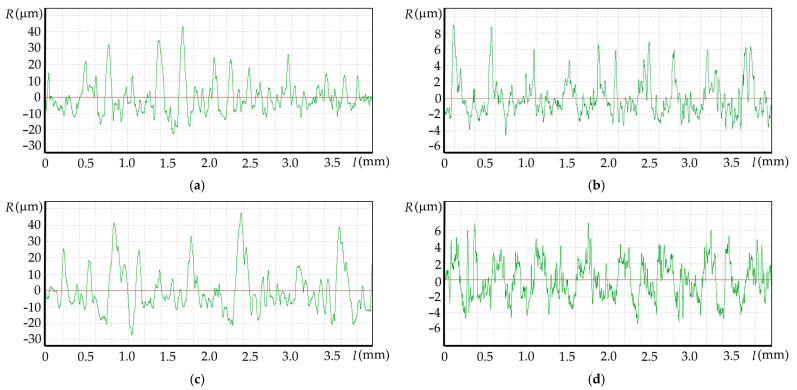
Measured surface roughness profiles: (**a**) PHA sample and feed rate of 0.05 mm/t; (**b**) PHA sample and feed rate of 0.13 mm/t; (**c**) PHA 2 sample and feed rate of 0.05 mm/t; (**d**) PHA 2 sample and feed rate of 0.13 mm/t.

Increased values of the Ra parameter were observed on surfaces made as a result of using the lowest analyzed feed rate, equal to 0.05 mm/t. The highest chip temperature values were observed when cutting at this feed rate. Surface roughness profiles obtained from measurements are burdened with anomalies that cause the parameter values to increase Ra. Observed anomalies consist of non-systematic peaks with a height ranging from 20 to 50 µm in the surface roughness profiles. They occur in both down-cut and up-cut milling areas. This observation suggests a significant correlation between chip temperature and the occurrence of defects. In order to demonstrate this relationship, Pearson correlation coefficient was calculated for the chip temperature values, and the Ra surface roughness parameter was obtained as a result of the measurements. The calculated Pearson correlation coefficients were r = 0.76 and r = 0.81 for areas milled in up- and down-kinematics, respectively. This indicates a strong positive correlation between the analyzed results. Examples of anomaly profiles obtained at a feed rate of 0.05 mm/t and obtained at feed rates of 0.13 mm/t without anomalies are shown in Figure 10a–d.

**Figure 11:**
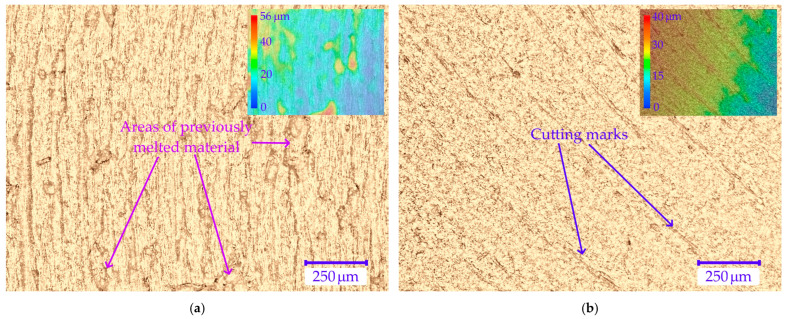

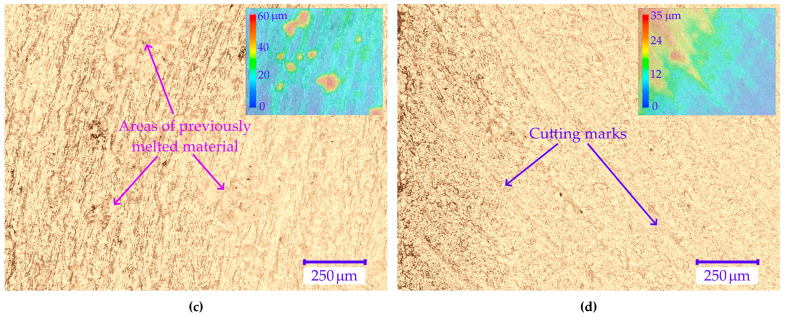
Images of samples surfaces taken using an optical microscope: (**a**) PHA sample and feed rate of 0.05 mm/t; (**b**) PHA sample and feed rate of 0.13 mm/t; (**c**) PHA 2 sample and feed rate of 0.05 mm/t; (**d**) PHA 2 sample and feed rate of 0.13 mm/t.

Observed anomalies result from high temperature in the cutting zone. Surfaces made at a feed rate of 0.05 mm/t are characterized by the presence of solidified, melted surface of the workpiece material during cutting. On the surfaces made at a feed rate of 0.05 mm/t, there are no machining marks typical of the milling process visible on the samples made at higher feed rates. Microscopic images of samples made using feed rates of 0.05 mm/t and 0.13 mm/t and diatomite contents of 0 and 2% are shown in Figure 11a–d. The images also show a view with a mask illustrating the height of the points on the sample surface.

## 4. Conclusions

In this study, the machinability of polyhydroxyalkanoate (PHA) with the content of diatomaceous earth was analyzed. Polyhydroxyalkanoates belong to the group of aliphatic polyesters and biodegradable materials of natural origin. Diatomaceous earth is a natural sedimentary rock composed of the shells of microscopic algae—diatoms. It is widely used as a filler in the plastics industry. Diatomite and polyhydroxyalkanoate are also biocompatible materials, making them widely used in tissue engineering and implants. The specific shapes of components for these applications makes machining more cost-effective and efficient in some cases than designing a single mold. Using biodegradable plastics with fillers of natural origin to produce machine parts is environmentally friendly by reducing waste and avoiding long-term soil and water pollution.

In the first stage, the samples of PHA, PHA 0.5, PHA 1, and PHA 2 were produced via injection molding with 0.5%, 1%, and 2% diatomite, respectively. In the next stage, the samples were milled. A diamond-coated carbide tool was used for this purpose. A groove was milled using the full width. The study analyzed the effect of feed rate and diatomite content in samples on chip temperature, the value of feed component of cutting force, and the roughness of resulting surfaces. Microscopic observations were also performed to analyze the prepared surfaces. As a result of this research, the following main conclusions were formulated:

1. It was shown that both from a statistical and technological point of view, analyzed diatomite contents in the processed samples do not have a significant impact on the machining process.
2. Excessive heat generated during the machining process can lead to surface melting of material, resulting in some finished surfaces being characterized by a heterogeneous structure and high roughness.
3. Increasing the feed rate to the maximum analyzed range (f_z_ = 0.13 mm/t) has a positive effect on the surface roughness and process efficiency. This is a consequence of reducing the heat generated by friction.
4. As a result of milling, a number of high-quality surfaces (Ra < 2 μm), chip temperature up to about 90 °C, and the value of the feed component of total cutting force below 11N were obtained.

Machining biodegradable materials belonging to the group of Green Polymeric Materials (GPM) is a modern field of mechanical engineering. According to the authors’ current knowledge, the presented study is the first to analyze the machinability of components made from biodegradable polymer materials with organic fillers.

Nevertheless, this study has some limitations. The research methodology based on experimental studies is susceptible to the influence of local machining conditions and the characteristics of the used machine tool. Cutting parameters such as cutting speed, depth, and tool wear were not analyzed. In addition, the tests were performed using a single type of cutting tool. In the future, it would be worthwhile to expand the range of machining parameters, consider different tools and coatings, analyze tool wear, and apply advanced statistical methods and process modeling. In addition, chip morphology analysis should be included to better evaluate the cutting mechanism and determine the heat balance in the cutting zone. This will provide a better understanding of energy distribution and the effect of diatomite additive on heat generation. It will also be important to conduct research under industrial conditions.

Low roughness, cutting forces, and temperature in the cutting zone ensure that our composites are characterized by high quality in the final product, including dimensional and shape compatibility. Obtaining such products allows for use in very demanding applications. There was no significant impact of machining on the final product properties, and the biodegradability of polyhydroxyalkanoates and diatomite also increase their potential for use in tissue engineering, among other applications.

## Figures and Tables

**Table 1:** Injection molding parameters.

Parameter	Value
Back pressure (bar)	90
Compression section (°C)	165
Convey section (°C)	160
Feed throat (°C)	21
Material temperature (°C)	160
Maximum moisture content (%)	0.025
Mold temperature (°C)	20–25
Nozzle temperature (°C)	170
Screw speed (rpm)	50

**Table 2:** Characterization of samples including chemical composition.

Sample	Composition (wt. %)
PHA	Pure polyhydroxyalcanoates
PHA 0.5	Polyhydroxyalcanoates with 0.5% of diatomite
PHA 1	Polyhydroxyalcanoates with 1.0% of diatomite
PHA 2	Polyhydroxyalcanoates with 2.0% of diatomite

**Table 3:** Parameters of used tools.

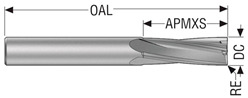							
DC (mm)	OAL (mm)	APMXS (mm)	RE (mm)	Tooth Number	Helix Angle (°)	Rake Angle (°)	Coating
12	100	36	0.2	4	10	0	DURA

**Table 4:** Tested feed values and diatomite content in produced samples.

Parameter	Tested Values
Diatomite content in samples (wt. %)	0; 0.5; 1; 2
Feed per tooth (mm/t)	0.05; 0.09; 0.13

**Table 5:** PHA material parameters.

Parameter	Value
Flow index (g/10 min 190 °C/2.16 kg)	10–20
Tensile modulus (MPa)	3300
Charpy shock unnotched (kJ/m^2^)	5
Temperature resistance (°C, HDT B)	121
Density (g/cm^3^)	1.25

## Data Availability

The original contributions presented in this study are included in the article. Further inquiries can be directed to the corresponding author.

## Appendix A

**Table A1:** The ANOVA results for chip temperatures.

Source	Seq SS	DF	MS	F-Value	*p*-Value
Diatomite content in samples	0.996	1	0.996	0.123	0.728 *
Feed per tooth, f_z_	1668.1	1	1668.1	205.5	0.001
Residual error	365.3	45	8.12		
Total	2034.4	47			

Seq SS—the sum of squares due to the source. DF—the degrees of freedom in the source. MS—the mean sum of squares due to the source. F-Value—the variance of the group means. *p*-Value—the probability that the difference between groups is due to chance. The * indicates non-important factors with a statistically non-significant influence on the measurement results.

**Table A2:** The ANOVA results for feed component of the total cutting force.

Source	Seq SS	DF	MS	F-Value	*p*-Value
Diatomite content in samples	0.782	1	0.782	3.77	0.058 *
Feed per tooth, f_z_	63.8	1	63.8	307.7	0.001
Residual error	9.33	45	0.207		
Total	73.9	47			

Seq SS—the sum of squares due to the source. DF—the degrees of freedom in the source. MS—the mean sum of squares due to the source. F-Value—the variance of the group means. *p*-Value—the probability that the difference between groups is due to chance. The * indicates non-important factors with a statistically non-significant influence on the measurement results.

**Table A3:** The ANOVA results for surface roughness, up-milling area.

Source	Seq SS	DF	MS	F-Value	*p*-Value
Diatomite content in samples	0.739	1	0.739	1.14	0.288 *
Feed per tooth, f_z_	177.8	1	177.8	274.8	0.001
Residual error	60.2	93	0.647		
Total	238.7	95			

Seq SS—the sum of squares due to the source. DF—the degrees of freedom in the source. MS—the mean sum of squares due to the source. F-Value—the variance of the group means. *p*-Value—the probability that the difference between groups is due to chance. The * indicates non-important factors with a statistically non-significant influence on the measurement results.

**Table A4:** The ANOVA results for surface roughness, down-milling area.

Source	Seq SS	DF	MS	F-Value	*p*-Value
Diatomite content in samples	7.49	1	7.49	8.38	0.005
Feed per tooth, f_z_	173.5	1	173.5	194.1	0.001
Residual Error	83.2	93	0.894		
Total	264.2	95			

Seq SS—the sum of squares due to the source. DF—the degrees of freedom in the source. MS—the mean sum of squares due to the source. F-Value—the variance of the group means. *p*-Value—the probability that the difference between groups is due to chance.
